# The symptoms of postpartum depression observed by family members: A pilot study

**DOI:** 10.3389/fpsyt.2022.897175

**Published:** 2022-10-13

**Authors:** Huong Thi Thanh Nguyen, Ly Thi Kim Do, Huong Thi Thu Pham, Anh Phuong Hoang, Hoa Thi Truong, Huyen Thi Hoa Nguyen

**Affiliations:** ^1^College of Health Sciences, VinUniversity, Hanoi, Vietnam; ^2^General Surgical Department, University Medical Center Schleswig-Holstein, Lübeck, Germany; ^3^Faculty of Nursing and Midwifery, Hanoi Medical University, Hanoi, Vietnam; ^4^School of Nursing, College of Medicine, National Taiwan University, Taipei, Taiwan; ^5^National Institute of Mental Health, Bach Mai Hospital, Hanoi, Vietnam

**Keywords:** symptoms, postpartum depression, role of family members, postpartum, family role attitudes

## Abstract

Postpartum Depression (PPD) is a burden on women's mental health after delivery, predominantly occurring in the 1st year. PPD poses a threat to the mother's life and affects the quality of childcare. Early detection by family members of depressive symptoms is critical. This study aimed to examine the role of family members in reporting depressive symptoms of PPD among new mothers. A cross-sectional study was conducted, where 56 family members were asked to report depressive symptoms observed in new mothers. At the same time, the new mothers were also screened for PPD using the Edinburgh Postpartum Depression Scale (EPDS). Binary logistic regression was performed. Depressive symptoms of new mothers reported by family members, including emotional and behavioral disturbance, being under stress, high anxiety, isolation, changing lifestyle, and inability to take care of their children, were found as predictors of PPD.

## Background

Postpartum Depression (PPD) is a major maternal health problem. The prevalence of PPD ranges from 1.9 to 82.2% in developing countries and from 5.3 to 74% in developed countries (1). In Viet Nam, a study discovered that the prevalence of women suffering from PPD was 20.4% in urban areas and 15.8% in rural areas (2). PPD is associated with a reduction in women's physical and mental health, and depressed women experience a lower quality of life (3). If a mother is depressed, anxious, or stressed, her children are more likely to have a wide range of adverse outcomes, including emotional problems, attention deficit hyperactivity disorder (ADHD), or impaired cognitive development (4). Social and/or family support plays a significant role in detecting PPD. When women receive support and care from their husbands, the percentage of those with depression reduces significantly (5). However, limited studies are available on the role of family members in screening for PPD. The sooner the PPD is detected, the better the outcome is achieved. This study, therefore, aims to explore the feasibility of family members of women with PPD helping detect early symptoms of PPD.

## Methods

This study is cross-sectional, piloted in a small group of families with new mothers whose child anywhere from birth to 1 year old. A convenience sampling method was used. The research team conducted home visits with new mothers whose children were under 1 year old to invite them to participate in this study. A brief introduction of this study and a consent form was sent out to all new mothers who gave birth within one previous year during the home visits in a commune of Thuong Tin district, Hanoi. In case mothers were not available at home during visiting time, the researchers would leave a message and return within that day. If the new mothers agreed to participate in the study, they were asked to complete the Edinburgh Postpartum Depression Scale (EPDS). The EPDS includes 10 items measured on a Likert scale of 0–3. A score of 12 and above indicates the risk of depression. The sensitivity and specificity of EPDS were 65–100% and 49–100% respectively (6). The validated Vietnamese version of EPDS is the most common screening tool for perinatal common mental disorders used in Vietnam with an internal consistency of 0.77 (7).

During home visits, researchers paid attention to the living environments and family members involved in caring for new mothers to identify the closest caregiver to that mother. The family members were asked to complete the second part of the questionnaire with 9 items to report any depressive symptoms they could observe in the new mothers. This part of the questionnaire was developed by the research team based on guidelines in DSM-V, including common items relating to the possibility of observing signs and symptoms of PPD among these new mothers (8). The evidenced-based items were developed based on DSM-V by the American Psychiatric Association and previous studies (5, 9, 10).

Although 116 families were reached out in total, only 56 families responded with both the answers of the new mothers and her family members.

SPSS software version 23.0 was employed for data management and analysis. Binary logistic regression was also performed to investigate the relationship between PPD among new mothers and depressive symptoms observed by family members. The proposal, including ethical considerations, was approved by Hanoi Medical University Research Proposal Committee, Decision No 5042/Q-HYHN.

## Results

Fifty-six new mothers participated in the screening with EPDS, and 20 (35.7%) of them were detected to be at risk of PPD (with an EPDS score of 12 and above). A family member of these mothers was also invited to an interview to report depressive symptoms observed from the mothers. Of the 56 family members, husbands were the majority (*n* = 29, accounting for 51.8%), followed by relatives and close friends (*n* = 17, accounting for 30.4%). There were only 5 maternal parents (8.9%) and 5 parents-in-law (8.9%) that participated in the interview (Table 1).

**Table 1:** The percentage of family members participating in the study.

**Family member**	**Number of participants (n)**	**Percentage (%)**
Husbands	29	51.8
Maternal parents	5	8.9
Parents in law	5	8.9
Relatives and close friends	17	30.4
Total	56	100

The relationships between observed depressive symptoms by family members and the risk of PPD among new mothers were reported. The results (Table 2) indicated that many symptoms and signs of PPD could be observed by family members. “Being sad unreasonably” was the most prevalent sign of PPD. As shown below, new mothers whom family members observed with the symptom “being sad unreasonably” experienced the risk of PPD (EPDS ≥ 12) 11.3 times higher than that of a new mother without this symptom (OR = 11.3; *p* < 0.05). Other significant depressive symptoms/signs observed by family members included “Separated to the outside” (OR = 8.8; *p* < 0.05); “Changing lifestyle” (OR = 7.6; *p* < 0.05); “Under stress and anxiety” (OR = 6.1; *p* < 0.05); “Uncontrolled emotion and behavior” (OR = 5.3; *p* < 0.05); and “Over-taking care of and being concerned about the child(ren)” (OR = ; *p* < 0.05).

**Table 2:** Depression symptoms reported by family members.

**Symptoms reported by a family member**	**EPDS**		**OR**
	**≥12**	**<12**	
Uncontrolling emotion and behavior	Yes	15	13	5.3^*^
	No	5	23	Reference
Being sad unreasonably	Yes	17	12	11.3^*^
	No	3	24	Reference
Under stress and anxiety	Yes	11	6	6.1^*^
	No	9	30	Reference
Separated to the outside	Yes	4	1	8.8^*^
	No	16	35	Reference
Changing lifestyle	Yes	11	5	7.6^*^
	No	9	31	Reference
Over-taking care of and concerning about the child(ren)	Yes	11	8	4.3^*^
	No	9	28	Reference
Ignoring child	Yes	1	0	2.9
	No	19	36	Reference
Having illusion	Yes	1	0	2.9^*^
	No	19	36	Reference

* p ≤ 0.05.

## Discussion

Depressive symptoms/ signs observed by family members in our study were consistent with the literature on signs and symptoms of PPD, including sleep disturbances, emotional disorders, and separating from society (2, 11, 12). Emotional and behavioral disturbance, being under stress and anxiety, isolation, changing lifestyle, and inability to take care of their child(ren) are likely predictors of PPD. These are similar to studies summarizing PPD psychosocial predictors such as stress, social support, and family connection (13), or factors of food intake patterns, sleep status, exercise, and physical activities (14) were also commonly reported. An explanation could be that the biological changes after delivery lead to fatigue and changes in emotions, behaviors, and daily activities (15). However, without early detection and/or proper treatment and care, psychoses occur in 1 to 2 per 1,000 postpartum women, and they may present as schizophrenic or affective disorders or as confused states (16).

Changing lifestyles reported by family members, such as eating habits or sleeping patterns, which were significantly associated with the risk of PPD, were also commonly found in women with PPD in other previous studies. It is not denied that we frequently observed the signs or symptoms of eating disorders related to depression among postpartum women. Typically, our results found a clear association between PPD and changing lifestyles (*p* < 0.001). Similarly, in another study, taking unhealthy food, and performing an unhealthy lifestyle, were found to have a significant relationship with depression, with 26.1% changing their daily lifestyle (17). Sleeping disorder is not only a predictor of PPD but also is a consequence of increasing PPD (18). Recently researchers examined the links between maternal sleep, maternal depressive symptoms, and mothers' perceptions of their emotional relationship with their infant in a self-recruited sample of mothers (11). Some studies described the association between serotonin and anxiety and depressive symptoms would be consistent with numerous observations indicating abnormal functioning of the serotoninergic system in depression for people experiencing anorexia or overeating (19).

## Conclusion

A larger-scale study with a bigger sample size is recommended to provide more substantial evidence that many symptoms/ signs of PPD can be reported by family members. It will then be followed by strategies to raise awareness that family members can play a crucial role in early screening for PPD.

## Data availability statement

The raw data supporting the conclusions of this article will be made available by the authors, without undue reservation.

## Ethics statement

The studies involving human participants were reviewed and approved by Hanoi Medical University Research Proposal Committee, Decision No. 5042/Q-HYHN. Written informed consent for participation was not required for this study in accordance with the national legislation and the institutional requirements.

## Author contributions

All authors listed have made a substantial, direct, and intellectual contribution to the work and approved it for publication.

## Conflict of interest

The authors declare that the research was conducted in the absence of any commercial or financial relationships that could be construed as a potential conflict of interest.

## Publisher's note

All claims expressed in this article are solely those of the authors and do not necessarily represent those of their affiliated organizations, or those of the publisher, the editors and the reviewers. Any product that may be evaluated in this article, or claim that may be made by its manufacturer, is not guaranteed or endorsed by the publisher.

## Abbreviations

PPD: postpartum depression
EPDS: Edinburgh Postpartum Depression Scale
DSM-V: Diagnostic and Statistical Manual of Mental Disorder V.
